# Cultural competency among Lithuanian nurses and preparedness to work with intercultural immigrants: A quantitative study protocol

**DOI:** 10.3389/fpubh.2022.1025508

**Published:** 2022-11-14

**Authors:** Rabie Adel El Arab, Rita Urbanavice, Agne Jakavonyte-Akstiniene, Marija Skvarcevskaja, Donatas Austys, Jose Tomas Mateos, Erica Briones-Vozmediano, Esther Rubinat-Arnaldo, Natalja Istomina

**Affiliations:** ^1^Faculty of Nursing and Physiotherapy, University of Lleida, Lleida, Spain; ^2^Healthcare Research Group (GRECS), Institute for Biomedical Research (IRBLleida), Lleida, Spain; ^3^Health and Social Services for Asylum Seekers Research Group, Vilnius University, Vilnius, Lithuania; ^4^Department of Nursing, Faculty of Medicine, Institute of Health Sciences, Vilnius University, Vilnius, Lithuania

**Keywords:** nurses, cultural competency, migrants, Lithuania, culture

## Abstract

**Introduction:**

Health care providers are increasingly required to provide care to patients from diverse cultural backgrounds. A culturally competent approach could be used to address gaps in the health care of migrants, whether they are refugees, asylum seekers, or undocumented migrants. From June 2021 onward, there are estimated to be 4,300 asylum seekers in Lithuania who crossed the Belarusian border. Furthermore, ~65 thousand Ukrainians registered within 6 months of the beginning of the war on 24 February 2022.

**Aim:**

To determine the cultural competence of Lithuanian nurses using the Nurse Cultural Competence Scale (NCCS) questionnaire.

**Methods:**

A quantitative study evaluating the cultural competency of nursing professionals will be conducted using the Lithuanian version of the Nurse Cultural Competence Scale (NCCS). The study will be conducted in Lithuanian municipalities and will involve primary, secondary, and tertiary health care providers.

**Discussion:**

This study will provide data that can guide the development and evaluation of interventions designed to reduce health disparities among migrants, including the need to identify the appropriate type of cultural competency training for nurses. In addition to the results of this study, it may provide an indication of other cultural competency required for nurses. This includes consideration of religion, sexual orientation, gender identity, household classifications on the basis of urban vs. rural areas, language spoken, and country of origin.

## Introduction

More than one million migrants and refugees entered Europe in 2015 (1). As a result, there were disagreements within the EU regarding the solution to this issue (1). The Lithuanian government pledged in 2015 to accept 1,077 refugees from EU and non-EU countries. During the year 2020, six refugees were transferred from Greece and Jordan to Lithuania. This agreement resulted in 499 people being resettled in Lithuania from 2015 to 2020 (2). Lithuania granted asylum to 80 individuals in 2020, a significant majority of whom were Russians (2). It is estimated that Lithuania received an unprecedented number of irregular immigrants who crossed the Belarusian border in June 2021. As a result, Lithuania is estimated to have around 4,300 irregularimmigrants in the country (3). Immigrants from the Middle East (Iraq and Syria) represent the majority of these individuals. In total, 2,858 migrants came from Iraq, which ranked first on the list of countries of origin for migrants. These were followed by 203 individuals who originated from the Congo and 179 individuals who originated from Syria (3).

As a result of the Russian invasion of Ukraine on February 24, 2022, more than 4.6 million refugees were forced to flee the country in just 2 months (4). As of October 2022, UNHCR statistics indicate that over 7 million refugees have registered throughout Europe, and ~4 million have been granted temporary protection within the EU (4).

In addition to this, there are over 6.5 million internally displaced persons in the country (5). As the Ukrainian government requires men aged 18–60 to remain and possibly participate in the war effort, mostly women, children, and elderly men enter the EU (6). It is estimated by UNICEF that 2.5 million children have been internally displaced and two million children have fled the country (7). Lithuania's Migration Department estimates that 65 thousand Ukrainians registered within 6 months after the war began on 24 February 2022 (8). Ukrainians are required to register at a registration center upon arrival in Lithuania. There are five Registration Centers located in Vilnius,Alytus, Marijampole, Klaipeda, and Šiauliai, which provide shelter, food, and clothing, as well as medical care; those in need are then transferred to long-term housing (9).

Health care providers are required to provide care to patients who are culturally and linguistically diverse, and that number is growing. Language and cultural barriers pose an increasingly significant threat to patient safety in hospitals (10), therefore cultural competence is increasingly recognized as a crucial aspect of providing quality healthcare to culturally diverse populations (11). The concept of healthcare cultural competence encompasses the ability to understand how social and cultural factors influence the health beliefs and behaviors of patients, and to take these influences into account at many levels of a healthcare delivery system in order to promote quality care (12). The presence of cultural misunderstandings increases a healthcare provider's perception of his or her readiness to treat culturally diverse patients, as well as negative attitudes toward cross-cultural care (13).

In order to overcome different communication difficulties, the key is to gain an understanding of how society and culture impact illness, as well as to reflect on their own strengths and weaknesses when communicating with different populations (14). Interpretation services were used to overcome communication challenges both face-to-face and over the telephone (15–18). However, despite the importance of interpreting services in healthcare, patients were frequently referred to interpreters through a time-consuming process (17, 18). Obtaining interpreters increased the nursing staff's workload, particularly in emergency situations (16). Furthermore, male interpreters were frequently incompetent in understanding the needs of immigrant women receiving maternity care (15).

An individual's cultural and ethnic background has a significant impact on his or her behaviors, emotions, and lifestyle (19, 20). It is crucial for health care providers to be able to provide care that understands the cultural influences on beliefs and customs that represent the different aspects of health and illness that clients have (19, 20). Nurses in Lithuania need to be sensitive to the impact of culture on health, as the country experiences an influx of immigrants and refugees (3). Providing nursing care is crucial to easing human distress, promoting comfort, and aiding in the face of life's challenges. In addition, nurse advocates play an essential role in facilitating the access of immigrants to health and mental health services, removing language and cultural barriers, regardless of their status as migrants, refugees, or asylum seekers (21).

Nursing in the global community should focus on developing new and innovative ways to improve and integrate health and social care processes in order to meet refugees' holistic needs (21). In an increasingly multicultural society, healthcare providers need to be aware of their patients' cultural needs and provide culturally sensitive care (22). It is therefore critical for healthcare professionals to be culturally competent in order to provide high-quality, effective care to patients from diverse cultures (23). The nurses are at the forefront of the healthcare system, they can play a vital role in providing culturally sensitive care. Nursing professionals with high cultural competence have been reported to be better able to establish cross-cultural communication with their patients, enabling them to effectively assess their patients' needs, plan appropriate treatments, reduce healthcare disparities, and significantly improve patient outcomes (24). Culture plays an important role in the improvement of patient outcomes, yet the presence of cultural competence is found to be low to moderate in studies from Italy (25), Iran (26), and China (27). The cultural competency of nurses can be enhanced through cultural training (28).

### Aim

Based on adaptations of the NCCS, the purpose of the study is to assess the cultural competence of Lithuanian nurses in caring for patients with diverse cultural backgrounds.

### Adaptation procedure

The purpose of the study is to assess the cultural competence of Lithuanian nurses using the Nurse Cultural Competence Scale (NCCS) questionnaire of S. Perng and R. Watson (29) who have given their written consent for its use and translation in Lithuanian. In addition, they agree to the use of the original scale as a Supplementary material in this article (Annex I). The scale was adapted according to the guidelines provided for the translation-back translation instruments procedure (30). Language adaptations of the scale have been documented at every stage. As part of the adaptation process of the original version, three bilingual healthcare researchers translated the NCCS into Lithuanian. In the next step, a panel of experts comprised of nurses with various professional backgrounds and roles developed version 1.0 of the Lithuanian NCCS scale. This translation was then converted into English by three bilingual healthcare researchers.

The translation from Lithuanian to English was then compared with the original English questionnaire. It was determined that the wording of items compared fairly on the basis of their significance. The pilot study was carried out by 20 master's degree nursing students at Vilnius University. The pilot study assessed the quality of translation, the appropriate cultural adaptation, and the feasibility of implementing the instrument.

Furthermore, the researchers were able to determine the time required to complete the questionnaire (i.e., 20 min). During a 3 focus group discussions with pilot study participants, an interviewer who is a co-author of the document discussed the comprehensibility and clarity of the survey items (Annex II). A variety of factors are considered, including religion, sexual orientation, gender identity, urban vs. rural housing, language spoken, and country of origin. Additionally, the participants were invited to contribute written suggestions for enhancing the readability of the scale items as well as for improving the graphic structure of the scale. Some changes were made.

### Instrument

The Lithuanian version of the Nurse Cultural Competence Scale (NCCS) will be used to evaluate the cultural competence of nursing professionals. The original NCCS consists of 41 items, which are divided into four subscales: Cultural Awareness (NCCS-CA)-10 items, Cultural Knowledge (NCCS-CK-)-9 items, Cultural Sensitivity (NCCS-CSe)-8 items, and Cultural Skills (NCCS-CS)-14 items. On a Likert scale of 1–5, the respondent's responses are scored based on how strongly they agree or disagree with the indicated statements. The overall score falls between 41 and 205 points. The higher score indicates a greater level of cultural competency.

## Methodology

### Nursing in Lithuania

The number of nurses per 1,000 people in Lithuania in 2019 was 7.7, which is lower than the EU average of 8.4. Furthermore, Lithuania has one of the highest numbers of doctors in the EU: 4.6 per 1,000, compared with the EU average of 3.9 (31). Although doctors are increasing in Lithuania, nurses are not, leaving 1.7 nurses per doctor in 2019—the lowest ratio since 2,000. There has been a decline in the number of nursing graduates since the 2000 s, from 626 in 2000–09 to 554 in 2010–19 (31). The aim of the National Health Strategy 2014–25 is to restore the nurse to doctor ratio to 2:1 (31). For the year 2020, statistics indicate that there are 21,906 nurses engaged in health care, health administration, health education, and research institutions (public and private) (32).

### Participants and data collection

The study will take place in the Lithuanian municipalities, in the premises of primary, secondary, and tertiary health care institutions. Nurses who are interested in participating in the study will be provisionally recruited. To qualify for inclusion, a nurse must be an active professional nurse, working full- or part-time in healthcare sector. In addition, nurses in academic or research institutions are excluded, and student nurses as well-because they are not formally recognized as registered nurses and their cultural competence is still developing as part of their university education. A questionnaire will be prepared in an electronic format and sent *via* official e-mails from the head nurses or administrators of health care institutions. Data will be collected from October to November 2022.

### Data analysis

The data will be entered and coded in Epidata version 4.6 for data entry and coding, and then exported to SPSS version 25 for analysis. The levels of cultural competency are determined according to the highest score achieved across the four subscales of the Nurse Cultural Competence Scale (NCCS). The Shapiro-Wilk test will be used to determine whether the variables are normally distributed. The *T*-test will be used to compare the means with the normal distribution of variables. When variables do not comply with the assumption of normality, the Mann-Whitney *U*-test will be used. The distribution of the respondents between the categories of categorical variables will be be compared using the χ2 test, if 20% or more of expected values are >5, and Fisher's Exact Test, if more than 20% of expected values are >5. The categorical variables will include gender (male/female), age (younger than median/older than median), and place of residence (urban/rural). The significance level for the statistical analysis will be 0.05. The measures of central tendency will be: mean ± standard deviation, for variables with a normal distribution, or median (first quartile-third quartile) for variables with a non-normal distribution.

## Discussion

The purpose of this study is to provide insights into the cultural competency of Lithuanian nurses. This study attempts to identify the characteristics, care situations, and nursing training associated with overall cultural competence. It is imperative that they demonstrate cultural competence if they are to be successful in their roles as nurses. Nurses will be able to rate themselves as somewhat culturally competent to culturally competent, moderately culturally competent, or as having low cultural competence. The study will yield data that can be used to guide the development and evaluation of interventions that can reduce health disparities in migrants, such as to determine the appropriate type(s) of cultural competency training that could help nurses achieve the goal of cultural competency. A culturally competent approach to care may be enhanced by formal courses, continuing education, practical experiences, and academic programs offering increased awareness, sensitivity, and behaviors (33). Nursing schools, employers, governmental and non-governmental institutions can all contribute to a culturally competent nursing workforce.

It was challenging to select appropriate cultural assessment tools during the preparation of this study. A variety of instruments were identified as a means of measuring the cultural competence of nursing students and professionals, including Bernal and Froman's Cultural Self-Efficacy Scale (34), a cultural competency assessment for health care providers, including nurses (35), a Nursing Cultural Competence Scale (NCCS) (29), and the Cultural Diversity Questionnaire for Nurse Educators (CDQNE) which developed to assess the cultural competency of nurse educators (36). In addition, we believe that the focus on the cultural competence attributes of nurses rather than the perceptions of patients (immigrants) about their care or the quality of their health may constitute a limitation of our study, as well as these instruments.

Another challenge for this study is the lack of funding resources. It was discussed among the team members whether to use paper surveys or online surveys, but most members preferred to use online surveys for various reasons, including the lower cost and the shorter amount of time required to solicit participants to complete the questionnairAs the purpose of the study is to reach nurses in Lithuania in different municipalities, we will be required to spend considerable time gathering data in the form of paper from each Lithuanian municipality and healthcare organization, as well as travel costs to various municipalities. As a result of the online format, we are probably going to receive fewer participants in the online surveys than if the team members were personally present at the health care institutions and invited nurses to participate in paper format. Further in the online survey respondents with biases may select themselves as part of the sample.

## Ethical considerations

The Declaration of Helsinki (37) has guided this study. This study complies with Lithuania's guidelines for biomedical and health research involving humans (2017). The Vilnius University Department of Nursing Ethics Committee approved this study (150000-KP-47). Consent will also be obtained from the healthcare institutions that will participate in the study. Researchers have developed an information sheet (Annex III) as well as a consent form (Annex IV) in English, which has been translated into Lithuanian. The participants will be informed that their participation in this research is voluntary and that their responses will be keptconfidential. In addition, they will be informed that they may withdraw from the study at any time.

## Dissemination plan

In this study, the dissemination plan is to disseminate the results, and additional data will be published in scientific journals and presented at scientific conferences and workshops.

## Patient and public involvement

Patients and the public will not be involved in the design of, recruitment for, and conduct of this study. Nurses will be invited to complete the nurse cultural competence scale in paper form.

## Author contributions

All authors have agreed on the final version and meet at least one of the following criteria [recommended by the ICMJE (http://www.icmje.org/recommendations/)]: (1) Substantial contributions to the conception or design of the work, or the acquisition, analysis, or interpretation of data for the work, (2) Drafting the work or revising it critically for important intellectual content, (3) Final approval of the version to be published, and (4) Agreement to be accountable for all aspects of the work in ensuring that questions related to the accuracy or integrity of any part of the work are appropriately investigated and resolved.

## Conflict of interest

The authors declare that the research was conducted in the absence of any commercial or financial relationships that could be construed as a potential conflict of interest.

## Publisher's note

All claims expressed in this article are solely those of the authors and do not necessarily represent those of their affiliated organizations, or those of the publisher, the editors and the reviewers. Any product that may be evaluated in this article, or claim that may be made by its manufacturer, is not guaranteed or endorsed by the publisher.
