# Supplementary material for: Cultural competency among Lithuanian nurses and preparedness to work with intercultural immigrants: A quantitative study protocol
Source: Front Public Health. 2022 Nov 14;10:1025508. doi: 10.3389/fpubh.2022.1025508 (PMC9702516; doi:10.3389/fpubh.2022.1025508)
Supplement: Supplementary file 1 [file Data_Sheet_1.docx]

**Annex I: Nurse Cultural Competence Scale of S. Perng and R. Watson 2012 (31)**

| Cultural Awareness Scale  What do you think about the following descriptions:  0 strongly disagree  1 disagree  2 no comment  3 agree  4 strongly agree | |
| --- | --- |
| One’s belief and behavior are influenced by one’s cultural background. | 0 1 2 3 4 |
| Those who came from diverse cultural backgrounds usually have different value systems. | 0 1 2 3 4 |
| Most people’s belief/behavior about health and illness are influenced by cultural values. | 0 1 2 3 4 |
| Understanding the client’s cultural background is very important to nursing care. | 0 1 2 3 4 |
| When getting immersed into a different culture, the acceptance level among individuals is quite different. | 0 1 2 3 4 |
| A client’s behavioral response originates from his/her cultural system, therefore the care provider should understand the client’s subjective interpretation of his/her own behavior. | 0 1 2 3 4 |
| Nursing education is itself a cultural system. | 0 1 2 3 4 |
| Understanding a client’s cultural background can promote the quality of nursing care. | 0 1 2 3 4 |
| A nurse’s cognition of health and illness is deeply influenced by nursing education. | 0 1 2 3 4 |
| Nursing knowledge and the client’s comprehension of interpretation of health/illness are usually different systems. | 0 1 2 3 4 |

| Cultural Knowledge Scale  What do you think about the following descriptions:  0 strongly disagree  1 disagree  2 no comment  3 agree  4 strongly agree | |
| --- | --- |
| I understand the social and cultural factors that influence health and illness. | 0 1 2 3 4 |
| I can identify the specific health problems among diverse groups. | 0 1 2 3 4 |
| I can use examples to illustrate communication skills with clients of diverse cultural backgrounds. | 0 1 2 3 4 |
| I can comprehend diverse cultural groups’ interpretations of their health beliefs/behavior. | 0 1 2 3 4 |
| I can list the methods or ways of collecting health-, illness-, and cultural-related information. | 0 1 2 3 4 |
| I am familiar in health- or illness-related cultural knowledge or theory. | 0 1 2 3 4 |
| I can explain the possible relationships between the health/illness beliefs and culture of the clients. | 0 1 2 3 4 |
| I can compare the health or illness beliefs among clients with diverse cultural background. | 0 1 2 3 4 |
| I can easily identify the care needs of clients with diverse cultural backgrounds. | 0 1 2 3 4 |

| Cultural Sensitivity Scale  What do you think about the following descriptions:  0 strongly disagree  1 disagree  2 no comment  3 agree  4 strongly agree | |
| --- | --- |
| I very much appreciate the diversities among different cultures. | 0 1 2 3 4 |
| I think it doesn’t matter what method of health s/he adopts, if has its advantages. | 0 1 2 3 4 |
| I can tolerate diverse cultural groups’ beliefs or behavior about health/illness behavior. | 0 1 2 3 4 |
| Even if a client’s use or adoption of a health maintenance method differs from my professional knowledge, I usually don’t appose it. | 0 1 2 3 4 |
| Even if a client’s use or adoption of a treatment method differs from my professional knowledge, I usually don’t prohibit it. | 0 1 2 3 4 |
| I usually discuss differences between the client’s health beliefs/behavior and nursing knowledge with each client. | 0 1 2 3 4 |
| I usually actively strive to understand the beliefs of different cultural groups. | 0 1 2 3 4 |
| In addition to traditional Chinese medicine and western medical ways of treatment, I would also try to understand alternative treatment methods. | 0 1 2 3 4 |

| Cultural Skills Scale  What do you think about the following descriptions:  0 strongly disagree  1 disagree  2 no comment  3 agree  4 strongly agree | |
| --- | --- |
| I can use communication skills with clients of different cultural backgrounds. | 0 1 2 3 4 |
| I can illustrate non-verbal expressions of clients from different cultural backgrounds. | 0 1 2 3 4 |
| Before planning a nursing activity, I will completely collect cultural background information on each client. | 0 1 2 3 4 |
| To me collecting information on each client’s beliefs/behavior about health/illness is very easy. | 0 1 2 3 4 |
| I can explain the influence of culture on a client’s beliefs/behavior about health/illness. | 0 1 2 3 4 |
| I can explain the influences of cultural factors on one’s beliefs/behavior towards health/illness to clients from diverse ethnic groups. | 0 1 2 3 4 |
| I can establish nursing goals according each client’s cultural background. | 0 1 2 3 4 |
| When implementing nursing activities, I can fulfill the needs of clients from diverse cultural backgrounds. | 0 1 2 3 4 |
| When caring for clients from different cultural backgrounds, my behavioral response usually will not differ much from the client’s cultural norms. | 0 1 2 3 4 |
| I can teach and guide other nursing colleagues about the differences and similarities of diverse cultures. | 0 1 2 3 4 |
| I can teach and guide other nursing colleagues about the cultural knowledge of health and illness. | 0 1 2 3 4 |
| I can teach and guide other nursing colleagues about the communication skills for clients from diverse cultural backgrounds. | 0 1 2 3 4 |
| I can teach and guide other nursing colleagues about planning nursing interventions for clients from diverse cultural backgrounds. | 0 1 2 3 4 |
| I can teach and guide other nursing colleagues to display appropriate behavior, when they implement nursing care for clients from diverse cultural groups. | 0 1 2 3 4 |

**Annex II: Interview guide, pilot study**

The FGDs will take place in a meeting room at a school of nursing. In accordance with COVID-19 precautions, chairs and tables will be arranged so that everyone can see and hear each other.

During this study, two tape recorders will be placed on a table so that each respondent is within equal distance from the other.

Respondents are informed of the purpose of the pilot study at the beginning of the FGD session. Before the actual FGD begins, participants will be informed that the FGD will be recorded, and they will be asked to provide their written consent.

The roles of the moderator and research assistant are described. It is important to emphasize that the conversation is confidential, and all concern and opinions, are highly appreciated.

A social opening will precede the FGD, in which participants will be addressed and thanked for participating in the pilot study. The introductory question will be **"We will discuss today the challenges associated with answering the Lithuanian version of the Nurse Competence Scale".**

Main Questions:

What are the challenges you faced when answering the different domains of the scale?

Follow-up questions such as, ‘you mentioned (- - -), could you explain what this means to you?’

Has anyone else experienced something similar?  Does anyone have a different perspective?

During the FGD, the moderator will keep track of the time and close the session at the end of the 60-minute session, or earlier if there are no new concerns that emerge, and everyone feels they have shared their perspectives of the translated scale.

In closing, the moderator will summarize what has been discussed throughout the session, and then ask if anyone has anything to add or correct, make a conclusion, or ask me any questions about the scale.

The participants are thanked for attending. After the FGD sessions, participants are invited to provide written feedback.

**Annex III: Information sheet about a research project: survey**

**Project title: Cultural competency among Lithuanian nurses and preparedness to work with intercultural immigrants**

**Introduction**

The faculty of medicine at Vilnius University would like to invite you to participate in a research study. Please read the following information carefully and discuss it with others, if you wish. Before making any decision, it is essential to know why the research is being conducted and what follows. If you have any questions, feel free to ask. You will be asked to fill a questionnaire if you decide to participate in this study. Withdrawal is possible at any time without giving reasons.

**What are the objectives of the study?**

There has been a significant increase in the number of migrants in Lithuania over the last few years. Those migrants may have different cultural backgrounds and speak different languages.  We distribute customized questionnaires to Lithuanian nurses to assess their cultural competence.

**Do I have to participate?**

Participation is completely voluntary and anonymous. It is entirely up to you whether you want to participate or not. If you choose to participate, you will be provided with a number of papers containing questions related to the research project (questionnaire). The expected time to fill out the questionnaire is about half an hour

**Is my participation in this study confidential?**

The study follows the Helsinki Declaration in the Ethics of Scientific Research, and the Ethics Committee of Vilnius University approved the study. Your participation in this study will be kept strictly confidential. No name or contact information will be necessary (anonymous). Vilnius University will keep all electronic information in password-protected files. Access to this information will be restricted to the research team only

**What are the advantages and disadvantages of participation?**

In this study, you will be able to determine your level of cultural knowledge and awareness. The study will provide valuable insight into how Lithuanian nurses are prepared to work with immigrants and provide high-quality care to all patients. As per academic research standards, there is no financial compensation for study participants.

**What will happen to the results of the research study?**

The results of this study will be published in academic journals, and the results will be shared with relevant governmental and non-governmental organizations in order to improve theweaknesses, as well as their participation in scientific conferences and symposiums at the national and international levels. You are not personally identifiable from any of these publications and results, as mentioned earlier. The results will be available to the participants in these surveys as well.

**Annex IV Consent to participate in the survey**

I agree to participate in a study entitled "**Cultural competency among Lithuanian nurses and preparedness to work with intercultural immigrants**."

I have agreed to participate in this research by completing the questionnaire provided by the research team. I am participating in this study voluntarily, and I have been informed that I can withdraw at any time during the study.

I have read the research information sheet. I have had the opportunity to ask questions regarding it. My questions were adequately addressed.

I agree to participate in the study voluntarily

Participant name

Participant's signature

Date

day / month / year
